# Optimizing Wheat Milling By-Products: An Overview of Processing Techniques

**DOI:** 10.3390/foods15061085

**Published:** 2026-03-20

**Authors:** Reham Ahmed Khashaba, Haiwei Lou, Yue Li, Saeed Hamid Saeed Omer, Xunda Wang, Zhonghua Gu, Renyong Zhao

**Affiliations:** 1 College of Food Science and Engineering, Henan University of Technology, Zhengzhou 450001, China; reham.khashaba2012@gmail.com (R.A.K.);; 2 Faculty of Agriculture, New Valley University, E1 Kharga, Kharga 72511, Egypt; 3 Food Laboratory of Zhongyuan, Luohe 462300, China

**Keywords:** wheat milling by-product, nonthermal treatments, nutritional modification, functional development

## Abstract

The increasing demand for novel and healthy food options is largely driven by the rise in lifestyle diseases and the global challenges of climate change. Annually, wheat by-products (WBP) production surpasses 150 million tons, with an anticipated growth of 10 million tons per year from 2021 to 2027. This surge has attracted researchers’ interest in leveraging WBP as sustainable food resources that promote human health. This review evaluates the effects of thermal and emerging nonthermal processing technologies on WBP, focusing on enzyme activity, antinutritional factors, bioactive compounds, antioxidant activity, and functional properties. Notably, thermal degradation poses significant challenges due to the heat sensitivity of WBP’s nutritional components. Therefore, nonthermal techniques like high-intensity ultrasound, radiofrequency, and cold plasma are being explored for their potential to enhance nutritional quality and extend shelf life. Further investigation is crucial to comprehensively understand the effects of these innovative treatments on WBP. Such research could facilitate the incorporation of treated WBP into the food industry, leading to new health-promoting products.

## 1. Introduction

The global grain mill products market is projected to reach 830.8 billion dollars by 2026, with wheat constituting approximately 84.5% of the total grain milled worldwide [1,2]. Annually, over 150 million tons of wheat by-products (WBP) are produced, with an anticipated growth rate of 10 million tons from 2021 to 2027 [3,4]. The three components of wheat are separated during the milling process: endosperm (83%), bran (14%), and germ (3%) [5]. The endosperm is rich in carbohydrates (76%), while wheat bran (WB) and wheat germ (WG) comprise 64% and 51% carbohydrates, respectively [6]. Contrary to starchy endosperm, dietary fiber dominates the carbohydrate content of wheat milling by-products (WBP), and most nutritional functions are linked to dietary fiber content [7]. Moreover, WBP is rich in protein content, and WBP proteins have much greater diversity and nutritional quality than the endosperm [8]. The most common WBP proteins are albumins and globulins, whereas the most common endosperm proteins are gliadins and glutenins [9]. As for lipid content, WBP has the highest lipid concentrations in wheat grains [10]. Polyunsaturated fatty acids represent the major WBP-lipid, up to 80% of the total triglycerides of WBP. The polyunsaturated fatty acid content indicates the high nutritional value of WB [11]. Also, Phenolic compounds are the most abundant bioactive compounds in wheat grains [12]. According to the literature, the content of phenolic compounds is 15- to 18-fold higher in WBP than in the endosperm [13,14]. Phenolic compounds are the main contributor to the antioxidant activities of WBP Therefore, WBP is superior to white wheat flour in terms of nutritional quality [15].

**Figure 1:**
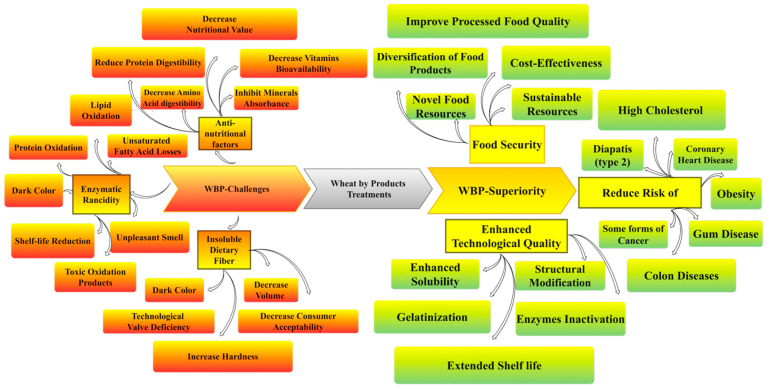
Challenges and superiority of using wheat by products (WBP) for human consumption and the food industry.

However, most wheat grain enzymes are found in the bran and germ, including lipase, lipoxygenase, esters, polyphenol oxidase, and peroxidase [16]. Enzyme activity leads to the hydrolytic rancidity of lipids, yielding free fatty acids [17,18,19,20]. Consequently, the free fatty acids are oxidized by lipoxygenase, which causes oxidative rancidity during storage [21]. Thus, enzyme activity decreases shelf life and leads to a decline in the sensory and nutritional properties of WBP. Phenolic compounds are widely recognized for their numerous health benefits; however, recent research indicates that certain high-molecular-weight polyphenols could pose challenges as antinutritional factors [22]. WBP-phenolics contain bound phenolic compounds such as phytic acid, tannins, saponins, and trypsin inhibitors [23]. These compounds have the potential to form strong, insoluble protein complexes, which may impede protein digestibility and nutrient availability, thereby ultimately diminishing the bioavailability of crucial minerals and vitamins [24]. Insoluble dietary fibers have some adverse effects on the technological functions of WBP. Therefore, WBP is mainly used for livestock feed and biogas production [25]. Figure 1 illustrates the challenges and superiority of using WBP for human consumption and the food industry.

Enzyme inactivation is one of the most crucial aspects of utilizing WBP in the food industry [26]. Consequently, thermal treatment strategies target decreased water activity in the enzyme environment or within the protein fraction, primarily reducing enzyme mobility [27]. Thermal treatments include dry heating (hot-air oven, microwave, and infrared) and wet heating (autoclaving treatment, steam explosion, and extrusion)]. However, thermal treatments impart many adverse effects on the nutritional and functional quality of WBP [28]. Therefore, numerous studies on the impact of emerging nonthermal treatments on preventing the adverse effects of thermal treatments have been conducted, such as high-intensity ultrasound [29,30,31,32], radiofrequency [33,34], and cold plasma [35,36,37,38]. Therefore, researchers are constantly trying to apply several thermal and nonthermal treatments to prolong the shelf life of WBP and enhance its nutritional and functional quality. Hence, this review article explores recent WBP thermal and nonthermal treatment research. Also, the review systematically shows changes in bioactive compounds, enzyme activity, antioxidant activity, and functional quality of WBP resulting from thermal and nonthermal treatments. The goal is to comprehensively evaluate the effects of these treatments on the nutritional and functional properties of WBP and provide a basis for their improvement. Hence, utilizing and exploiting WBP is promoted as part of healthy and sustainable food production worldwide during food manufacturing.

## 2. Nutritional Value and Bioactive Contents of Wheat-Milling By-Products

Wheat-milling by-products are highly nutritious and contain various bioactive compounds, such as dietary fibers, biogenic amines, and phenolic compounds [39]. WBP accounts for about 74% of the total bioactive compounds in wheat grains, with bran and germ contributing 50% and 23%, respectively [40,41]. The main dietary fiber components are arabinoxylan and β-glucan, which constitute 70% and 20% of the total dietary fiber content in WBP, respectively [42]. Arabinoxylans contain phenolic acids like ferulic acid, covalently bound to xyloses or arabinoses. Feruloylated arabinoxylans have drawn the attention of the pharmaceutical sector due to their anti-cancer, prebiotic, and antioxidant properties [43]. *β*-glucan is efficient in trapping reactive oxygen [44,45,46]. *β*-glucan lowers the glycemic index, and blood sugar, low-density lipoprotein cholesterol (LDL), along with the antioxidant, anticancer, and free radical scavenging properties.

The significance of dietary fiber for human health cannot be overstated; it supports gastrointestinal function, offers diverse antioxidant properties, and boosts the immune system [5,40,47]. WBP stands as a remarkable source of soluble dietary fiber, boasting an impressive 46% non-starch polysaccharides [48]. Among these, arabinoxylan leads the way at 70%, followed by beta-glucan. It has been reported that soluble dietary fiber reduces overall intestinal enzymatic activity, lowering postprandial plasma glucose levels [49,50]. The synthesis of short-chain fatty acids (SCFAs), which have a significant role in the control of cardiovascular diseases (CVDs), is also increased by soluble dietary fiber owing to their high fermentability [51]. On the other hand, WBP stands out as an exceptional source of insoluble fiber, boasting a powerful combination of cellulose, hemicellulose, and lignin [41]. Insoluble dietary fiber, primarily acts as a laxative and bulking agent, increasing fecal mass or shortening intestinal transit time [52]. Enhanced satiety or decreased body weight could also be linked to an insoluble dietary fiber mechanism for managing non-communicable diseases (NCDs) [53]. Soluble and insoluble dietary fiber relieves constipation, lessens bile salt reabsorption, and reduces the risk of colon cancer [54,55].

**Figure 2:**
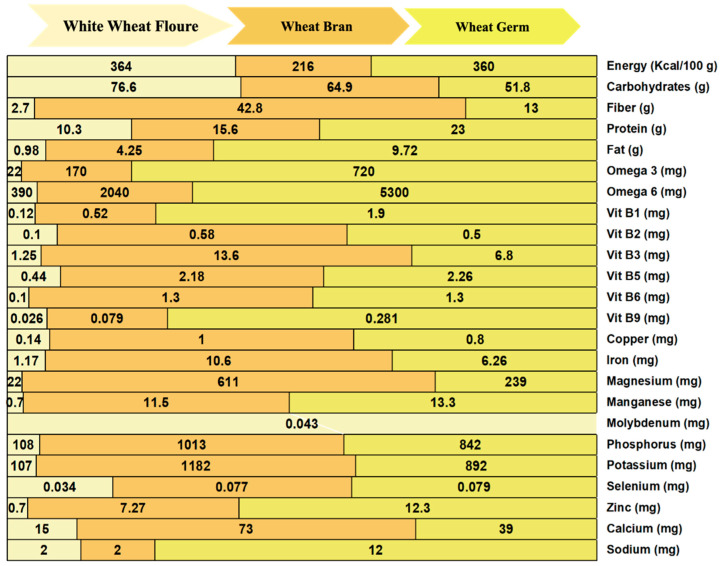
Comparing macronutrients and micronutrients per 100 g of white wheat flours, wheat bran (WB), and wheat germ (WG).

Furthermore, WBP represents an astounding 83% of the total phenolic content found in whole wheat grains [56]. Their antioxidant prowess is particularly noteworthy, driven by a high *α*-tocopherol content of 57% and an impressive *γ*-tocopherol proportion of 39%, surpassing sunflower and olive oils [57]. Moreover, WBP proteins demonstrate high digestibility and a balanced amino acid profile, making them a valuable source of amino acids such as alanine, arginine, asparagine, and glycine [58]. Essential amino acids, including histidine, valine, lysine, and threonine, are also present in significant amounts in WBP proteins [59,60]. The digestible amino acid value (DIAAS) for leucine and isoleucine in WBP are 82% and 93%, respectively. In comparison, milk whey protein exhibits DIAAS values of 1.72 for leucine and 1.97 for isoleucine [6]. The biological value of the proteins in WBP (23%), rivaled that of animal proteins found in meat (16–22%) and milk (3.5%), WBP proteins have the potential to produce bioactive peptides, which indicates their suitability for human nutrition [61]. Furthermore, over 85% of wheat grain micronutrient content is concentrated in the WBP [62,63,64,65,66]. Figure 2 compares macronutrients and micronutrients in white wheat flour, WB, and WG. WB and WG contain approximately 77–80 µg/100 g selenium, respectively, significantly surpassing the daily human requirement of 55 µg. The daily human requirements for iron, manganese, and phosphorus are 8 mg, 300–400 mg, and 700 mg, respectively. Remarkably, 100 g of WBP can fulfill the daily human needs for iron, manganese, and phosphorus, as illustrated in Figure 2 [48,67,68,69]. WBP is one of the few natural plant parts considered a rich source of vitamin B-complex except for vitamin B12. Among B complex vitamins, Vitamin B3 (14.85 mg/100 g) is the predominant vitamin in WBP [70,71,72,73].

WBP-bioactive compounds impart their health benefits through multifaceted physiologic mechanisms, including facilitating substance transit through the digestive tract, butyric acid production in the colon, food absorption and dilution in the intestine, antioxidant activity, and immune system enhancement [57,74]. Notably, incorporating indigestible dietary fibers and phytochemicals found in WBP can lead to reduced peripheral insulin resistance and improved glucose mobility [75,76,77], while also promoting a healthier inflammatory response healthier [78,79,80]. Embracing WBP bioactive compounds can be a transformative step towards better health.

The summary of WBP’s contribution to human health is presented in Table 1. This summary is backed by strong evidence from clinical studies that have evaluated the relationship between WBP consumption and a reduced incidence of hypercholesterolemia, atherosclerosis, cardiovascular diseases [81], type 2 diabetes [82], and different types of cancer [83,84]. Also, systematic reviews have been published summarizing WBP benefits and confirming that WBP consumption within the human diet could boost the immune system and consequently could positively decrease the higher risk of experiencing frequent infections and lifestyle diseases [25,57,85,86,87,88,89,90,91,92,93,94]. Recognizing the exceptional nutritional properties of WBP, various innovative thermal and nonthermal processing techniques have been developed to enhance its shelf life. It is crucial to investigate how these treatments may alter the nutritional and technological quality of WBP, determining whether the resulting changes are advantageous or detrimental. The subsequent sections will present insightful discussions about the impacts of these processes on enzyme activity, bioactive compounds, antioxidant activity, and the overall functional quality of WBP, emphasizing the need to prioritize this powerful food source in our diets.

## 3. Effects of Thermal Processing Technologies on Wheat Milling By-Products

Thermal treatments involve direct heat transfer through dry or hydrothermal treatments, resulting in moisture loss, structural changes, and the formation of complex flavor compounds [18,103]. Dry heat treatment of WBP demands heat transfer through conduction and convection (hot air oven) or radiation (microwave and infrared heating). In contrast, wet thermal treatment requires heat transfer through the steam (autoclaving, superheated steam, and steam explosion treatments) or wet thermal treatment by mechanical-assisted extrusion. Table 2 shows the impact of thermal treatments on WBP quality.

### 3.1. Hot Air Oven (Conventional Dry Heating)

A hot air oven is considered the most widely employed WBP treatment. During the hot air oven treatment, the WBP is typically spread out in a thin layer on trays or conveyor belts and exposed to controlled heat. Given that WBP is exposed to high temperatures for a long time during hot air oven treatment, hot air oven treatments have various effects on the functional properties of WBP [125]. Protein solubility is an important functional property of WBP, as it influences its applicability in various food and industrial formulations. Hot air oven treatments could affect protein solubility by denaturing or modifying the structure of proteins. Higher temperatures and longer treatment times typically result in more significant protein denaturation, leading to reduced solubility.

Several studies have been conducted to assess the impact of hot air oven treatments on enzyme activity and the shelf-life prolongation of WBP [104,105,106,107,126]. Arslan et al. [105] found that treating WG at 160 °C for 6 min in a hot air oven reduced residual lipoxygenase and lipase activity by 3% and 15%, respectively. In a separate study by Meriles et al. [106] WG treated at 175 °C for 20 min in a convective oven had low residual enzyme activity of lipoxygenase (1.33%) and lipase (6.71%). This study suggested that lipase is more stable than lipoxygenase under dry heat treatment. Abdel-Haleem. [104] reported that lipase enzymes in whole grain bread are partially stable against denaturation under hot air oven treatment compared to WG. They found that treating WB at 175 °C for 20 min effectively reduced lipase activity by half, while WG showed a complete loss of lipase activity. This difference may be attributed to WB’s drier environment than WG’s.

A recent study by Erim investigated the impact of hot air oven treatment on WG at different temperatures (120, 130, 140, 150, and 160 °C) and durations (0, 5, 10, 15, and 20 min) [107]. The study confirmed that lipoxygenase is more sensitive to temperature in the convection oven at 120 °C than lipase. It concluded that hot air oven treatments above 120 °C for 20 min caused a significant loss of phenolic compounds in WBP [107]. The reduction in total phenolic compounds after hot air oven treatments may be due to oxidative and thermal degradation. These findings suggest that conventional hot air treatments are not favorable for maintaining the nutritional and functional quality of WBP. Additionally, hot air oven treatments are associated with high energy consumption and environmental sustainability concerns, indicating the need for alternative processing methods or technologies to improve efficiency and sustainability.

### 3.2. Microwave and Infrared Heating

Microwave and infrared heating are alternative methods of dry heating that offer rapid heating compared to traditional hot air oven heating [127,128,129]. Li et al. [109] investigated the effect of short-wave infrared radiation (100 watts) at 90 °C for 20 min on WG. They reported that the residual lipase activity in WG was 18.02%. However, they also observed that the high temperature generated during the treatment resulted in the degradation of several bioactive compounds in the WG. Conversely, Gili et al. [110] found that infrared at an intensity of (4800 W/m^2^) for 3 min was more effective for achieving heat penetration and inactivating enzymes while maintaining the bioactive compounds in the WG. They also found that the treated WG exhibited an extended shelf life of at least 90 days when stored at controlled room temperature in sealed packages, compared to the untreated WG, which had a shelf life of approximately 15 days. These findings suggest that employing high-intensity short-wave infrared treatment might offer a promising solution for enzymatic inactivation and shelf-life extension of WBP while retaining their valuable bioactive compounds.

Microwave heating is known for its ability to generate heat rapidly within a product through molecular motion, which leads to various effects on nutrient bioavailability. Arslan et al. found that treating WG with a low-power microwave at 180 W (2450 MHz) for 12 min caused a reduction in the phenolic compounds and antioxidant activity [105]. Conversely, the higher microwave power resulted in shorter treatment times, which helped prevent the thermal degradation of bioactive compounds in WBP. However, Liu et al. found that the total phenolic compounds increased by 37%. The antioxidant activity increased from 3.43 to 14.76 mg/100 g after treating WB by microwave heating at 7.5 kW for 2 min [114]. They reported that prolonged treatment time (700 W for 5 min) would continue to reduce enzyme activities up to the maximum reduction of lipase by 65% and lipoxygenase by 99%.

However, the moisture content of WG decreased to 95%, which was deemed unsuitable for maintaining its properties. They concluded that microwave power at 7.5 kW for 2 min was the optimal treatment condition for enzyme inactivation in WB, which also resulted in improved nutritional quality. Similarly, Zhang et al. [111] recommended an optimal microwave treatment of 560 W for 3 min for enzyme inactivation in WG. The molecular docking results from Qu et al. [115] showed that the inactivation of lipase was due to the conformational changes in the lipase catalytic sites. The entrance of the catalytic cavity contracted, which made the fat substrate unable to enter the active cavity quickly as control, which prevented the fat hydrolysis.

On the other hand, Zhang et al. [111] found that several aromas, such as esters (fruity), acids (pungent), alcohols (green and aromatic), and alkanes (smoky), decreased or even disappeared as the microwave treatment at 560 W for 3 min. In contrast, some other compounds emerged or increased, such as nitrogen-containing compounds with roasted and coffee-like aromas, aldehydes (almond-like), ketones (coffee-like), and heterocyclic compounds (cooked potato). Microwave heating can cause protein chains to unfold partially, which helps proteins interact with the air-water interface and improve absorption. The increased protein absorption can contribute to enhanced foaming capacity and foaming stability due to the formation of a thick proteinaceous film around air bubbles. The protein film acts as a protective layer, preventing the air bubbles from collapsing, ultimately leading to enhanced foam formation and stability [130]. The results reported by Lauková et al. supported these findings and provided additional confirmation of the optimal microwave treatment conditions for potential improvements in the properties of WBP [112,113]. They reported that microwave power at 800 W for 2 min could improve the hydration properties, modify the color parameters (lightness, yellowness), increase redness and chroma, and decrease hue angle, as well as potentially decrease the antinutritional agents. Hence, the literature suggests that infrared and microwaves could be treatments for enhancing grain properties and be used in large-scale industrial processing [131,132]. Further research could provide valuable insights and help boost the incorporation of WBP into the food industry by enhancing its functional properties and extending its shelf life.

### 3.3. Hydrothermal Treatments Without Mechanical Process (Autoclaving and Superheated Steam)

The critical aspect of hydrothermal treatment is the combination of high temperature and pressure in a liquid medium [2,108]. Hydrothermal treatments without mechanical processes are also effective for WBP treatments, including conventional hydrothermal (autoclaving) and superheated steam techniques. When WBP is exposed to high temperatures under wet and pressurized hydrothermal conditions, the steam condenses on the WBP surface. Once the WBP temperature reaches saturation, the moisture will evaporate from the WBP [133,134].

Many studies reported that hydrothermal treatment had a better effect than hot air oven treatments due to the double action of pressure and high temperature in the wet environment [108,134,135,136,137,138]. Rico et al. [116] evaluated the effect of autoclaving on the quality and techno-functionality of WB at different temperatures (100, 115, and 130 °C). They found that the autoclave treatment enhanced water absorption capacity and reduced WB pasting viscosity at 130 °C. This treatment resulted in an ingredient with high storage stability, antioxidant properties, a four-fold increase in the concentration of free ferulic acid (compared with non-treated WB), and an increased content of flavonoids. In this context, Hu et al. compared the effect of hot air oven treatment with hydrothermal treatment on WB enzyme activity at the same temperatures (170 °C). They found that the peroxidase, lipase, and lipoxygenase were entirely inactivated after 16 min of hot-air oven treatment and 7 min of superheated steam treatments [20]. These results are consistent with Arslan’s study reported that conventional hydrothermal treatment at 121 °C for 20 min (autoclave steaming) had a better enzyme inactivation effect than hot air oven treatments at 160 °C for 6 min in WG [105]. However, they found that autoclave steaming significantly reduced total tocopherols, which was directly associated with the more significant loss of *β*-tocopherol content. On the contrary, *γ*- and *δ*-tocopherol and tocotrienol homologs were more abundant, with higher amounts after autoclave steaming. *α*-Tocopherol and *γ*-tocotrienol were the most resistant isomers to the stabilization processes.

Since superheated steam forms at a temperature that exceeds saturated steam at the same pressure, superheated steam has a faster heating rate and higher efficiency than autoclaving treatments [139]. Furthermore, superheated steam has been reported to preserve unstable nutrients and bioactive compounds better than autoclaving treatments [128]. Hence, conventional wet thermal treatments have been developed to reduce treatment time, improve industrial production efficiency, and reduce expense by utilizing superheated steam treatment. Therefore, more research is needed to understand the impact of superheated steam treatment on the nutritional properties, functional characteristics, and overall quality of WBP.

### 3.4. Hydrothermal Treatments with the Mechanical Process (Steam Explosion and Extrusion)

Steam explosion treatment is considered a viable alternative to autoclaving as a hydrothermal treatment [140,141]. It offers advantages such as shorter treatment times and potential improvements in phytochemical contents and antioxidant activity. A study by Kong et al. [121] compared the effects of steam explosion treatment at 170 °C for 5 min with conventional autoclaving at 120 °C for 20 min on the properties of WB. They found that the steam explosion treatment was able to completely inactivate the enzymes in WB after only 5 min, whereas autoclaving required 20 min to achieve the same effect. Steam explosion and extrusion treatments have been shown to improve the hydration properties of WBP [117,140,142,143]. The high temperature and pressure applied during these treatments help loosen the structure of WBP, allowing it to absorb water more readily. Fourier transform infrared spectroscopy (FTIR) spectra and scanning electron microscopy microscope (SEM) results confirmed that a steam explosion had the effect of breaking the cell wall of WB due to the conversion of water-unextractable arabinoxylan (WU-AX) to water-extractable arabinoxylan (WE-AX) and the release of *β*-glucan [119,120,136]. Recent research has indicated that hydrothermal and pressure treatments, such as autoclaving and extrusion, significantly improve the hydration properties of WBP. Specifically, these methods enhance the water absorption index and water retention capacity when compared to conventional dry heating treatments. This evidence underscores the advantages of adopting these advanced techniques for improved performance in various applications [123,143,144,145]. Improving the hydration capacity is crucial for various food applications, including enhancing texture and retaining moisture in baked goods. Lee et al. [123] concluded that the extrusion had the best effect on WB’s water absorption index and water retention capacity, as well as bulk density and soluble dietary fiber, compared with autoclaving (135 °C for 5 min) and hot air oven (200 °C for 15 min) treatments. The moisture content during hydrothermal treatments with the mechanical process plays a crucial role in the polymerization of phenols and their antioxidant activity [134,139,146]. Kong et al. [119] reported that the concentration of total phenolics and flavonoids in WB increased by 50% and 35%, respectively, after steam explosion-assisted superfine grinding treatment. The high shear force and temperature during extrusion promote the release and reactivity of phenolic compounds, leading to improved functionality. Studies have reported positive effects of extrusion on phenolic compounds and the antioxidant activity of WB [120,123,147,148]. Ramos et al. [118] reported that the optimized extrusion conditions were 30% feed moisture and 140 °C final extrusion temperature using response surface methodology. Overall, hydrothermal treatments such as steam explosion and extrusion offer promising approaches to enhancing the nutritional and functional quality of WBP and similar by-products [134,144,146]. However, further research is needed to understand the role of extrusion and steam explosion in improving WBP comprehensively.

## 4. Effects of Emerging Nonthermal Processing Technologies on Wheat Milling By-Products

Most thermal treatments face disadvantages, such as high temperatures or extended processing times, low loading, limited flexibility, and expensive operating costs [149,150]. Hence, research groups have developed various nonthermal treatment techniques to meet thermal treatment challenges [151]. Nonthermal processing technologies are often considered more environmentally friendly compared to traditional thermal treatments [56,75]. They typically require less energy and have shorter processing times, reducing the overall carbon footprint [152,153]. Table 3 shows the impact of non-thermal treatments on WBP quality.

### 4.1. High-Intensity Ultrasound

Treating WBP with ultrasonic depends on a phenomenon known as acoustic cavitation. Wherein the movement of high-intensity ultrasonication (>1 W/cm^2^, 100–200 kHz) waves generate mechanical vibrations, resulting in the inception of gas/vapor bubbles (acoustic cavitation) [32]. The bubbles expand and then implode during the propagation of ultrasound waves, leading to a high temperature (upwards of 1000 °C) and pressures (50–500 MPa). This process occurs in a concise time domain of approximately one microsecond, and continuous cycles of compression and rarefaction are produced, causing a series of alternating contractions and expansions [59]. Consequently, the generation of high turbel turbulence causes shear forces that form microfractures in food components, which modify their structure and change their functional properties [169].

One of the significant effects of ultrasound treatment is its ability to induce mechanical, thermal, and chemical changes that affect the secondary and tertiary structures of enzymes, resulting in the loss of their biological activity [170]. Therefore, Habuš et al. [158] studied the effect of ultrasound treatment on WB. They reported that high-intensity ultrasound treatment with an amplitude of 80% for 15 min and 15% bran suspension reduces lipase activity by 64%, peroxidase by 90%, and polyphenol oxidase by up to 93%. Meanwhile, they studied the possibility of extending the oxidative stability of WB through *p*-anisidine value. They found that high-intensity ultrasonication could prolong the oxidative stability of WB for 12 months with the preservation of total phenolic content and the antioxidant activity of WB due to the reduction in enzyme activity. An excellent advantage of high-intensity ultrasound treatment is its ability to extend the shelf life of treated WBP while maintaining its antioxidant capacity.

### 4.2. Radiofrequency

Radiofrequency is a dielectric heating technique that has the same principles as microwaves [30,171]. The main difference is that radiofrequency (27.12 MHz) has a longer wavelength than microwaves (2.45 kHz), which leads to a deeper penetration and more uniform heating than microwave heating. When grain milling by-products are coupled with the electromagnetic wave inside the radio frequency cavity, the electromagnetic energy is converted into thermal energy by migrating polar water molecules and ionic components in grain milling by-products [171]. Hence, heat generates moisture evaporation from the grain milling by-product surface and stimulates moisture migration from the inside to the outside [172].

In this context, Ling et al. investigated the effect of a hot air-assisted radio-frequency heating system (6 kW, 27.12 MHz) on enzyme inactivation of WG [173,174]. They reported that treating WG with radio-frequency treatment at 100 °C for 15 min or with hot air heating at 110 °C for 5 min effectively inactivated lipase activity by 18.2% and 22.5%, respectively. After 120 days of treated WG storage, they found that lipase activity was significantly lower than untreated WG (*p* < 0.05). According to the free fatty acid value and peroxide value, they reported that the treated WG could be maintained in acceptable quality for more than 90 days of storage under controlled room conditions in a zip-lock PE bag. Ling et al. [155,175] compared radiofrequency as a nonthermal treatment with steaming as a thermal treatment for WG. They found that the total phenolic content was 456.1 mg GAE/100 g WG after the radiofrequency treatment while steaming decreased to 392.8 mg GAE/100 g WG. Also, they found higher retention in antioxidant activity after radiofrequency (21.1 μmol Trolox/g WG) compared to the steaming treatments (14.1 μmol Trolox/g WG). They also reported that the radiofrequency treatment improved the protein solubility, foaming, and emulsifying properties of WG more effectively than hydrothermal treatments. This was attributed to the fact that hydrothermal treatments cause severe protein denaturation and accumulation, which negatively affects these properties [176].

These results are consistent with the findings of another study by Liao et al. [156,157]; they reported that radiofrequency treatment (12 kW, 27.12 MHz) to 100 °C for 15 min holding at 100–105 °C or 110 °C with 6 min holding at 110–115 °C reduced lipase activity to about 10% in WG. Radiofrequency treatments disrupt the delicate balance of forces that maintain the protein structure of enzymes, leading to changes in the tertiary and quaternary structures and, thus, loss of the catalytic activity of enzymes [34,177]. The findings indicate that radiofrequency technology has the potential to effectively and economically enhance the nutritional and functional quality of WBP. Such non-thermal methods could serve as suitable substitutes for thermal treatments.

### 4.3. Nonthermal Plasma

Cold plasma is the fourth state of matter, classified as an ionized gas, following solid, liquid, and gas states. It is generated by intensifying electric and electromagnetic energy at low pressure in gases like oxygen, nitrogen, helium, neon, and argon, resulting in the creation of highly reactive ionized gases known as cold plasma [60,149,178,179]. Cold plasma treatment is a promising non-thermal technology that has been explored for various applications, including food processing [180]. It was observed that cold plasma treatment has the potential to enhance WBP functional properties and improve their nutritional profile [28]. It has been observed that cold plasma treatments can break peptide bonds, cause modifications to amino acid side chains, and cleave bonds within the *α*-helical structure of enzymes, leading to alterations in the three-dimensional structure [181,182]. These modifications in the secondary structure can ultimately alter the enzyme’s three-dimensional structure. Since the three-dimensional structure of the enzyme is crucial for its function, any disruption to this structure can potentially impact its activity [149]. In this context, cold plasma treatment at two different voltages (20 and 24 kV) on the inactivation of lipase and lipoxygenase of WG has been studied [102,154]. The findings demonstrated that after 25 min at a voltage of 24 kV, lipoxygenase and lipase were inactivated up to 75% and 50%, respectively. It was observed that treated WG by cold plasma (at 24 kV, 25 °C for 25 min) showed high retention of total phenolic content, and the DPPH radical scavenging activity did not change significantly compared to untreated WG. These results are consistent with another study by Tolouie et al. Additionally, Tolouie et al. compared cold plasma and steam autoclaving treatments of WG, reporting that steam autoclaving reduced antioxidant activity and total phenolic content by 14.70% and 30.80%, respectively [102]. Therefore, cold plasma treatments represent a promising nonthermal technology, as they offer rapid processing times at low temperatures, which help extend shelf life while preserving the nutritional compounds of WBP [183].

Cold plasma treatment has been shown to induce physical and structural changes in WBP, leading to enhanced functional properties in food applications [182]. Cold plasma treatment can increase the solubility and dispersibility of the WBP components [183]. Furthermore, it was reported that cold plasma treatment increased protein solubility, emulsifying properties, and foaming properties [184]. The increased solubility and dispersibility of the components offer several advantages in food applications. For example, they can enhance the texture, viscosity, and emulsifying properties of food products. Food products containing plasma-treated WBP may exhibit improved sensory attributes, such as smoother texture, better mouthfeel, and enhanced stability of emulsions [181,185].

It is important to note that cold plasma treatment has the potential to enhance both WB and WG. However, further research is required to fully understand its effects on various factors. Such as plasma power, treatment time, and gas composition, as well as how the specific characteristics of WBP may influence the outcomes of cold plasma treatment [186]. Additionally, sensory evaluation and consumer acceptance studies should be conducted to assess the impact of cold plasma treatment on the sensory attributes of wheat bran and wheat germ-based products. Research holds the key to unlocking the valorization mechanism of WBP through emerging nonthermal plasma treatments, as highlighted in Table 2. It is essential to consider the cost implications of large-scale industrial processing of cold plasma and other non-thermal treatments, paving the way for innovative advancements.

## 5. Challenges and Future Perspectives

Ohmic Heating, ultraviolet irradiation, and supercritical carbon dioxide are promising alternative nonthermal treatments for enhancing the nutritional and functional quality of grains [187,188,189,190]. Numerous studies have demonstrated that ohmic heating could significantly enhance the nutritional and functional quality of food materials when compared to traditional hot air oven treatments [191]. The inside-to-outside heating nature of ohmic heating resolves temperature lag issues obtained with traditional outside-to-inside heating methods, allowing high temperatures to be reached without burning the surface of WBP. Additionally, ohmic heating offers more than 95% energy efficiency and low maintenance costs.

In addition to ohmic heating, ultraviolet-C is based on the same principle as radiofrequency treatment (converting electromagnetic energy into thermal energy) [192]. During food treatments, ultraviolet-C is applied at 250 to 280 nm wavelength. Hence, it exhibits outstanding permeation capabilities to foodstuff without compromising nutritional and functional quality. Moreover, ultraviolet-C radiation is easy to utilize, cost-efficient, non-toxic, and environmentally friendly. Therefore, the nonthermal technologies of ultraviolet-C radiation and radiofrequency technology have been extensively studied in the food industry [193]. It was observed that ultraviolet irradiation has a potential capacity for enzyme inactivation in rice bran and prolongs its shelf life [194] due to its ability to disrupt the functionality and integrity of enzymes’ DNA. Also, high-pressure carbon dioxide is a nonthermal treatment method that involves applying pressurized carbon dioxide at pressures ranging from 1 to 500 bar. Most studies on high-pressure carbon dioxide have been conducted in the supercritical phase (pressure > 73.9 bar and temperate > 31.9 °C), in which carbon dioxide has density like liquid (0.9–1.0 × 10^3^ kg/m^3^), gas-like diffusivity, and viscosity (10^−7^–10^−8^ m^2^/s and 3–7 × 10^−5^ N s/m^2^, respectively), along with zero surface tension [187,195,196]. These properties enable supercritical carbon dioxide (scCO_2_) to penetrate the complex structures of food materials more efficiently. Since carbon dioxide is non-flammable, its critical temperature can easily reach 31 °C with pressures of 73.8 bar. The scCO_2_ is considered a convenient, large-scale, and low-cost technology [197]. Hence, supercritical carbon dioxide has gained significant attention as a promising nonthermal technology for food treatments [187,190,198]. Despite the promising potential of ohmic heating, ultraviolet irradiation, and supercritical carbon dioxide, their effects on the nutritional and functional quality of WBP have not yet been fully explored. These treatments still need further investigation [150,151,188,199,200,201].

Treated WBPs have shown potential in protecting against cancer, diabetes, heart disease, and other chronic conditions, while also aiding in weight management, according to epidemiological studies and emerging research. Although the results indicate an inverse relationship between treated WBP bioactive compounds (such as phenolic acids, flavonoids, and fibers) and health outcomes, some discrepancies in human intervention studies and mechanisms of action remain subjects of ongoing investigation. Further research is required to better understand how bioactive compounds from thermal and nonthermal-treated WBP affect gut microbiota and their mechanisms of action in humans. Future studies exploring the complex link between WBP treatments and health could significantly contribute to developing the next generation of nutritious WBP-based foods. Consumers are encouraged to incorporate a wider variety of WBP into their diets to promote health and reduce the risk of chronic diseases.

## 6. Conclusions

This review provides valuable reference data on the effects of both thermal and emerging nonthermal technology on the nutritional and functional quality of wheat milling by-products (WBP). The findings can be summarized into three main categories based on their impact on WBP: (1) enzyme inactivation, (2) effects on bioactive compounds, and (3) improvements in functional and technological properties. Emerging nonthermal treatments have demonstrated the potential to enhance the bioactive compounds in WBP, maintain their antioxidant properties, and extend their shelf life. Furthermore, nonthermal processing offers advantages such as shorter treatment times, higher energy efficiency, and improved safety compared to conventional thermal treatments. As a result, nonthermal technologies present an attractive solution for enzyme inactivation, shelf-life extension, and enhancing both the technological quality and nutritional value of WBP. Scaling up nonthermal processing from laboratory to industrial applications remains a challenge. Efficient, cost-effective equipment is needed to process large quantities of WB and other grains while maintaining consistent treatment parameters. Addressing these challenges will require continued research and technological advancements in nonthermal processing technologies. The insights gained from this review can help improve the treatment of WBP, contributing to more environmentally sustainable food production and health promotion. Moreover, these advancements could play a key role in supporting sustainable food systems to meet the needs of the growing global population (projected to reach 10 billion by 2050) by promoting resource recirculation in a circular economy.

## Figures and Tables

**Table 1:** Summary of the recent clinical studies related to WBP-contribution to human health.

Study Participants Sex, Baseline Age (Years)	WBP	Duration	Main Findings	References
31 adults (15 M and 16 F) Av age 32 years	48 g WB breakfast cereal	3 weeks	- Increase plasma ferulic acid.	[95]
12 adults (6 M and 6 F) Av age 24 years	4.9 g WB-arabinoxylan extract	Single test meal	- Enhances colonic bacterial metabolism. - Boosts fermentation and bacterial growth indicators.	[96]
40 adults Diabetic (higher insulin) Av age 29 years	60 g WB	1 year	- Increase plasma butyrate and acetate. - Increase short chain fatty acids production and GLP-1 secretion in hyperinsulinaemic humans. - Reduce risk for diabetes.	[97]
63 adults (33 M and 30 F) Av age 42 years	8 g WB-arabinoxylan extract in soft drink	3 weeks	- Increase short-chain fatty acids in feces by 8%. - Increase fecal propionic acid level. - Reduce protein fermentation. - Increase fecal bifidobacteria levels.	[98]
55 adults Av age 18–75 years	44 g WB daily	3 weeks	- Increases plasma ferulic acid. - Reduce LDL cholesterol.	[99]
29 adults Av age 19–44 years	10 g WB-arabinoxylan extract	3 weeks	- Increases colonic fermentation protein. - Selectively stimulate the growth of bifidobacteria and change fermentation in the colon.	[100]
19 adults (9 M and 10 F) Av age 23 years	8.9 g WB-arabinoxylan extract	Single test meal	- Improve insulin sensitivity index. - The capacity to impact overnight glycaemic regulation.	[54]
10 adults Av age 18–65 years	20 g WB	Single test meal	- Labeled fermentation markers appear in breath and plasma about 3.75 h after consumption and last for 8 h. - Increase plasma short-chain fatty acids for about 8 h.	[101]
8 healthy adults Av age 18–55 years	120 g WB breakfast cereal	Single test meal	- Increase plasma, urine, and fecal short chain fatty acids and butyrate. - Increase plasma ferulic acid by 5 h. - Regular consumption could support a healthy gut environment.	[91]
81 adults (49 M and 32 F) Av age 40–65 years	40 g WBP	6 weeks	- Increase bowel movement frequency and stool weight. - Increase in stool total short chain fatty acids and acetate.	[91]
8 healthy volunteers Av age 23 years	120 g WB	2 weeks	- Potential to deliver anti-inflammatory compounds directly into the lower gut. - Increase ferulic acid and reduce inflammations. - The management of inflammatory disorders in the liver. - Control of many metabolic disturbances. - Beneficial effects on physiological functions in the gut.	[78,91]
60 healthy volunteers	3.5 g WG oil	30 days	- Positive effect on the normalization of lipid metabolism. - Decrease in total cholesterol levels, triglycerides, and low-density lipoprotein levels. - Potentially for the prevention of cardiovascular diseases, atherosclerosis and obesity.	[88]
48 normoglycemic adults	15 g WB	12 weeks	- Increase fecal Bifidobacterium. - Stools softer without significant effects on energy metabolism in healthy humans with a gastrointestinal slow transit.	[90]
20 adults Av age 18–30 years	10 g WB	5 days	- Increase faecal bulk and improved laxation. - Potential role in relieving constipation: help improve gut function.	[85]
21 male and female Av age 60 years	15 g WB, three times daily	5 days	- Boost the levels of Bifidobacterium species. - Significant increase in fecal total short-chain fatty acids. - Promote the growth of bifidobacteria.	[86]
75 male and female	20 g WG	12 weeks	- Significant reduce stress and depression scores.	[102]
8 healthy volunteers Av age 18–55 years	40 g WB	Single test meal	- Increase colonic short-chain fatty acids levels from 96.88 to 136.96 mM. - Promote microbial butyrate formation. - Reduce colonic inflammation. - Increase plasma folate levels. - Support a-butyrogenic fermentation and gut health. - Potential role inhibiting colorectal adenomas.	[92]

**Table 2:** Effects of thermal treatments on the quality of wheat by products: wheat bran (WB) and wheat germ (WG).

WB/WG	Treatment/ Process	Effects	References
Thermal processing technologies (1-Conventional dry heating)			
WG WB	175 °C/20 min	- Reducing lipase activity by half in WB. - Reducing lipase activity by 100% in WG.	[104]
WG	160 °C/6 min	- Reduce enzyme activity. - Residual lipoxygenase 3%. - Residual lipase activity 15%. - Not providing an advantage in oxidative stability compared to autoclave steaming and microwave.	[105]
WG	175 °C/20 min	- Lipase is more stable than lipoxygenase. - Residual activity of lipase 1.33%. - Residual activity of lipoxygenase 6.71%. - Loss of protein solubility and formation of aggregates. - No effect on water-holding, oil-holding, and foaming capacity.	[106]
WG	120 °C/20 min	- −120 °C/20 min is the best hot air oven processing condition to stabilize the raw germ. - Reduce lipase activity by 87.29%.	[107]
WB	105 °C/40 min	- Increase phenolic acids. - Increase soluble dietary fiber. - Decrease insoluble and total dietary fiber. - Disrupted protein matrix, smooth surface, and small amounts of gaps by SEM.	[108]
WB	120 °C for 20 min	- Increase omega-6/omega-3 ratio. - Increase volatile compound content. - Effectively released WB phenolic compounds. - Enhanced antioxidant activity.	
Thermal processing technologies (2-Microwave and infrared heating)			
WG	Short wave infrared/ 100 W at 90 °C/20 min	- Residual activity of lipase 18.02%. - Residual activity of lipoxygenase 19.21%. - Peroxide value remained below 5% for 60 days. - No significant decrease in fatty acids. - Degradation of several bioactive compounds.	[109]
WG	Short wave infrared 4800 W/m^2^/3 min	- Maintaining the bioactive compounds. - Storage at room temperature in sealed packages for at least 90 days.	[110]
WG	Microwave/ 180 W/12 min	- Reduction in phenolic compounds and antioxidant activity.	[105]
WG	Microwave 560 W/3 min	- Improved nutritional quality. - Maintain the inherent colour. - Decrease the content of esters, alkanes, alcohols, and acids. - Increase heterocyclic compounds, nitrogen-containing compounds, aldehydes, and ketones content. - Increase compounds with a roasted flavour - Less volatiles with a grass-like flavour.	[111]
WB	Microwave 800 W/2 min	- Decrease antinutritional agent. - Reduce phytic acid content by 49%. - Improve the hydration properties. - Modify the colour parameters (lightness, yellowness), increase redness and chroma, and decrease hue angle.	[112,113]
WB	Microwave 7.5 kW/2 min	- Increase the total phenolic compounds by 37%. - Increase the antioxidant activity. - Reduce enzyme activities. - Improve nutritional and functional quality.	[114]
WB	Microwave 700 W/3 min	- Reduce lipase activity by 92%. - Retard rancidity. - Maintain the bran quality.	[115]
WB	Microwave 800 W/1.50 min	- Increase soluble dietary fiber. - Reduce insoluble and total dietary fiber - Gallic acid, p-hydroxybenzoic acid, ferulic acid, syringic acid, vanillic acid, *p*-coumaric acid increased by 12.02%, 29.46%, 69.75%, 145.12%, 56.80%, 106.22%, respectively.	[108]
WB	Microwave 7.5 kW for 120 s	- Increase omega-6/omega-3 ratio. - Increase volatile compound content. - Effectively released WB phenolic compounds. - Enhanced antioxidant activity.	[2]
Hydrothermal treatments (1-Autoclaving, superheated steam (without mechanical process))			
WB	Superheated steam 170 °C/ 7 min compared with hot air oven/ 20 min at the same temperature	- Superheated steam has better effects than the hot air oven. - Increase extractable phenolic compound contents. - Increase antioxidant activities. - Decrease peroxide value. - Increase unsaturated fatty acid contents. - Improve sensory evaluation scores.	[20]
WB	Autoclave 130 °C/3 min	- Enhance the antioxidant properties. - Increase free ferulic acid concentration. - Increased apigenin-6-C-arabinoside-8-C-hexoside content. - Enhanced water absorption capacity. - Reduce pasting viscosity. - Reduction the glycemic load.	[116]
WB	Autoclave 121 °C/90 min	- Increase phenolic acids. - Increase soluble dietary fiber. - Decrease insoluble and total dietary fiber. - The fiber structure exhibits a loose configuration characterized by small particles and irregular flakes.	[108]
Hydrothermal treatments (2-Steam explosion and extrusion (with the mechanical process))			
WB	Steam explosion and extrusion	- Improve the water-holding capacity and swelling capacity. - Reduce the lightness, sodium cholate, cation. exchange capacity and phytate content.	[117]
WB	Extrusion 120 °C, 30% feed moisture	- Increase bioactive compounds and antioxidant activity.	[118]
WB	Steam explosion 170 °C/5 min assisted superfine grinding	- Increase water solubility index. - Increase oil-holding capacity. - Increase bile salts and cholesterol adsorbing capacity - Increase total phenolic and total flavonoid content. - Show the most muscular DPPH radical scavenging activity.	[119]
WB	Extrusion 120 °C, 23% feed moisture, 310 rpm	- Increase water-holding capacity and extract viscosity. - Increase arabinoxylan and ferulic acid solubilisation. - Starch melting. - Phytate degradation.	[120]
WB	Steam Explosion at 0.8 MPa 170 °C/5 min compared with Autoclaving (0.1 MPa 121 °C/20 min	- Steam Explosion has better effects than autoclaving - Increase flavonoids by 198%. - Increase phenolic contents by 83%. - Increase soluble dietary by 27%. - Increase DPPH radical scavenging activity by 21%. - Inactivate enzyme activity ultimately. - Decrease fatty acid value by 21%. - Decrease peroxide value by 75%. - Inhibits rancidity. - Decrease insoluble dietary fibre content by 24%.	[121]
WB	Extrusion 160 °C, 17% feed moisture, 275 rpm	- Reduces lipase and polyphenol oxidase activity by 51% and 68%, respectively.	[122]
WB	Extrusion 120 °C, 26% feed moisture, 250 rpm.	- Increase the total phenolic content, bulk density, and soluble dietary fibre compared with autoclaving (135 °C for 5 min) and hot air oven (200 °C for 15 min) treatments. - Increased the hydration properties compared with dry heating treatment.	[123]
WB	Extrusion 110 °C, 25% feed moisture,140 rpm	- Increase ferulic acid, vanillin, and apigenin by 10-fold comparing raw wheat bran. - Improve antioxidant and anti-cancer activities.	[124]

**Table 3:** Effects of nonthermal treatments on the quality of wheat by products: wheat bran (WB) and wheat germ (WG).

WB/WG	Treatment/ Process	Effects	References
Emerging nonthermal processing technologies			
WG	Cold plasma, 20, 24 kV, 25 min	- Inactivation of 50% of lipase and 75% of lipoxygenase. - High retention of bioactive compounds. - Improve antioxidant activity. - Prolong the shelf life while maintaining the nutritional compounds.	[102,154]
WG	Radiofrequency 100 °C/15 min holding in hot air heating and 110 °C/5 min	- Increase bioactive compounds. - Improve antioxidant activity. - Improve the protein solubility, foaming, and emulsifying properties.	[155]
W G	Radio-frequency to 100 °C/15 min holding in hot air heating 110 °C/5 min	- Effectively inactivate lipase activity. - Decrease free fatty acid value. - Decrease peroxide value. - Acceptable quality for more than 90 days of storage.	[156,157]
WB	High-intensity ultrasound/ 15 min and 15% suspension	- Reduces lipase, peroxidase, and polyphenol oxidase activity by 64, 90, and 93%, respectively. - Preservation of total phenolic content. - Boost antioxidant activity. - Prolong the oxidative stability for 12 months.	[158]
WG	Cold plasma, 50 Hz, 24 kV, 35 min	- Cold plasma treatment has better effects than autoclaving. - The residual activity of lipase and lipoxygenase decrease up to 87.72 and 92.52%, respectively. - No significant difference during treatment duration of antioxidant potential and phenolic compounds.	[102]
WB	Microwave-assisted hydrolysis 7.5 kW for 120 s at controlled temperature (50–70 °C)	- It is a non-thermal treatment used to improve the quality of wheat bran. - Decrease the omega-6/omega-3 ratio compared to conventional - Treatments, and it showed the best ratio. - Showed the lowest volatile compound content compared to conventional treatments. - Showed higher bioaccessible phenolic compounds content (1168%) than raw WB. - It enhanced the bioavailability of phenolic compounds. - It improved the WB-matrix structure and nutritional and functional characteristics more effectively than conventional methods. - Increased ferulic acid up to 11 mg/g WB. - Recorded the highest antioxidant activity compared to raw WB and conventional treatments. - It demonstrates an increase of 118.45% in DPPH radical scavenging activity and 30.11% in ABTS radical scavenging activity compared to raw WB. - A combination of microwave and hydrolysis is a practical nonthermal approach to improve WB quality.	[2]
WB	Atmospheric plasma, 10 kV, 6 kHz and up to 24 kV, 50–Hz (5–35 min)	- Penetrated the WBP-caryopses and activated their physiological reactions. - Inactivates rancidity enzymes. - Enhances the shelf life. - No alterations in fat, protein, ash, and moisture content. - Enhancement of soluble protein content by 15%.	[159]
WB	Cold plasma 40 V, 0.9 ± 0.1 A for 2–6 min	- Improved its free amino acid profile. - Enhancing its nutritional value. - Improved surface color appeared brighter and yellower. - Gave off a pleasant mellow aroma, - Removing unpleasant flavor. - Decreased flavonoid and anthocyanin content. - Increase total phenolic content. - Improve the physicochemical properties and sensory quality.	[160]
WG	Atmospheric cold plasma at 25 kV for 5–40 min	- Increase protein solubility. - Improved emulsifying activity and stability, foaming capacity, and protein stability. - Plant-based protein modification. - Water holding capacity values were significantly increased by increasing processing time. - The oil holding capacity was significantly reduced by increasing the time.	[161]
WB	Ultrasound-assisted hydration 0–1500 W at 15–25 °C	- Improvement in hydration kinetics. - Increasing the temperature of the medium reduced the processing time up to 55% at a same acoustic power.	[162]
WB	Ultrasound 400 W for 1–10 min	- No statistically significant difference in total solid yield and total carbohydrate content. - No effect on the monosaccharide composition.	[30]
WB	Low-frequency ultrasound at 120 W- for 23 min	- Increase phenolic compounds extraction by 70% comparing to solvent extraction. - Increase in ferulic acid extraction. - Reduction in the enzymatic activities of lipase, lipoxygenase and polyphenol oxidase.	[163]
WB	Ultrasound (180 W) at 50 °C, for 70 min,	- Increase polysaccharides yield by 142.6 mg/g.	[164]
WB	Ultrasound 152 to 750 W	- Increased total phenolic content by 21.30%. - Enhance the release efficiency of valuable compounds.	[165]
WB	supercritical carbon dioxide at 25.0 ± 0.1 MPa and 40 ± 2 °C	- WB-oily extract was stable during 155 days of storage at 21 °C in darkness. - Increase bioactive compounds such as alkylresorcinols, steryl ferulates, tocopherols, and a small amount of other phenolic compounds. - Low levels of oxidation parameters (low hydroperoxides and hexanal content) and relatively high antioxidant global capacity.	[166]
WB	Cold plasma 50 V for 3 min ultrasonic 200 W for 3 min	- Cold plasma and ultrasonic treatments are operationally flexible, chemical-free, and sustainable for the preparation of polysaccharide-polyphenol conjugates. - Enhanced in vitro antioxidant activities. - Modifying the functional and nutritional quality of polysaccharides.	[167]
WB	Ultrasonication (500 W) and microwave (25 min) combination sodium hydroxide 0.30 mol/L	- Increase antioxidant activity and extraction yield of hemicelluloses and reduce the extraction time compared to conventional treatments. - Enhance extraction yield and purity.	[168]

## Data Availability

No new data were created or analyzed in this study. Data sharing is not applicable to this article.
